# Inhibiting Airway Smooth Muscle Contraction Using Pitavastatin: A Role for the Mevalonate Pathway in Regulating Cytoskeletal Proteins

**DOI:** 10.3389/fphar.2020.00469

**Published:** 2020-05-06

**Authors:** Robin A. Lu, Amir A. Zeki, Sumati Ram-Mohan, Nhan Nguyen, Yan Bai, Kenneth Chmiel, Stevan Pecic, Xingbin Ai, Ramaswamy Krishnan, Chandra C. Ghosh

**Affiliations:** ^1^Department of Emergency Medicine, Center for Vascular Biology Research, Beth Israel Deaconess Medical Center, Harvard Medical School, Boston, MA, United States; ^2^Division of Pulmonary, Critical Care, and Sleep Medicine, U.C. Davis Lung Center, University of California Davis School of Medicine, Sacramento, CA, United States; ^3^Pulmonary and Critical Care Medicine, Brigham and Women's Hospital, Harvard Medical School, Boston, MA, United States; ^4^Department of Chemistry and Biochemistry, California State University, Fullerton, CA, United States

**Keywords:** statin, bronchodilation, asthma, stretch, inflammation, mevalonate, mechanics, mechanopharmacology

## Abstract

Despite maximal use of currently available therapies, a significant number of asthma patients continue to experience severe, and sometimes life-threatening bronchoconstriction. To fill this therapeutic gap, we examined a potential role for the 3-hydroxy-3-methylglutaryl-coenzyme A reductase (HMGCR) inhibitor, pitavastatin. Using human airway smooth muscle (ASM) cells and murine precision-cut lung slices, we discovered that pitavastatin significantly inhibited basal-, histamine-, and methacholine (MCh)-induced ASM contraction. This occurred *via* reduction of myosin light chain 2 (MLC2) phosphorylation, and F-actin stress fiber density and distribution, in a mevalonate (MA)- and geranylgeranyl pyrophosphate (GGPP)-dependent manner. Pitavastatin also potentiated the ASM relaxing effect of a simulated deep breath, a beneficial effect that is notably absent with the β2-agonist, isoproterenol. Finally, pitavastatin attenuated ASM pro-inflammatory cytokine production in a GGPP-dependent manner. By targeting all three hallmark features of ASM dysfunction in asthma—contraction, failure to adequately relax in response to a deep breath, and inflammation—pitavastatin may represent a unique asthma therapeutic.

## Introduction

During an asthma exacerbation, airway smooth muscle (ASM) contraction is a primary end-effector of bronchoconstriction (Macklem, 1996; Gunst and Tang, 2000; An et al., 2007; Prakash, 2016). Importantly, ASM mass is substantially enhanced in severe (Benayoun et al., 2003) and fatal (Carroll et al., 1993) asthma, and its potential as a therapeutic target, is therefore, in principle, even greater in these sub-populations. However, current therapies directed at reducing ASM contraction, including β2-agonists and muscarinic antagonists, are inadequate in controlling asthma symptoms and/or exacerbations (National Asthma and Prevention, 2007; Peters et al., 2007; Chapman et al., 2008; Webb, 2011). In part, this is because existing therapies target ASM receptor-mediated pathways that are complex, indirect, and susceptible to desensitization (Whalen et al., 2007; Penn, 2008). Ideally, we need an asthma therapy that can directly inhibit the force-generating ASM contractile machinery. Such a strategy would also be preventative as it will limit the ability of the asthmatic airway to narrow.

To disrupt the ASM contractile apparatus, we considered a potential role for 3-hydroxy-3-methylglutaryl-coenzyme A reductase (HMGCR) inhibitors, also known as the statin drugs or “statins.” HMGCR is the rate-limiting enzyme of sterol biosynthesis in the mevalonate (MA) pathway. Supporting the relevance of this pathway are adjacent studies in endothelial cells, saphenous vein smooth muscle cells, and fibroblasts, that have revealed that lipophilic statins can inhibit the RhoA/ROCK pathway (Turner et al., 2005), MLC phosphorylation (Lampi et al., 2016), and F-actin levels (Farina et al., 2002; Chubinskiy-Nadezhdin et al., 2017). Although similar effects have never been directly reported in ASM, our previous work performed using a murine model of acute allergic airway inflammation has noted that some inhaled statins can reduce airway resistance and airway hyper-responsiveness (AHR), including airway hypersensitivity (Xu et al., 2012; Zeki et al., 2015; Wu et al., 2017). However, these measurements were performed using murine models of airway inflammation, and therefore, we cannot rule-out that statins' anti-inflammatory effects could have had a secondary (or indirect) beneficial effect on AHR.

Therefore, the question remains: can statins have an independent, therapeutic effect on airway mechanics *via* direct effects on ASM? To answer this question, we chose the moderately lipophilic statin, pitavastatin, with a previously noted anti-inflammatory effect e.g., (Yuan et al., 2012; Wu et al., 2017), and examined its ability to regulate ASM contraction and inflammation in cultured human ASM cells, and methacholine (MCh)-induced bronchoconstriction in murine precision cut lung slices (PCLS). Our key findings are: 1) pitavastatin blunts basal-, histamine-, and MCh-induced ASM contraction; 2) pitavastatin potentiates the ASM relaxation effect of a simulated deep breath; 3) pitavastatin inhibits ASM pro-inflammatory cytokine and chemokine secretion. These beneficial effects on ASM contraction occur by a MA- and geranylgeranylpyrophosphate (GGPP)-dependent mechanism that was further confirmed by gene silencing of HMGCR in ASM. Taken together, these data support further investigation into the use of pitavastatin as a novel therapy for alleviating ASM dysfunction in asthma.

## Materials and Methods

### Cell Culture

Primary human ASM cells that were previously generated from non-asthmatic and asthmatic donors (Comer et al., 2014), as per Panettieri *et al*. (Panettieri et al., 1989), were made available to us through a Material Transfer Agreement with the Gift of Hope Organ and Tissue Donor Network (Itasca, IL). These cells exhibit significant bronchodilator responsiveness (Yoshie et al., 2018). Cells were grown in 10% serum-containing F12 medium (Krishnan et al., 2008; Krishnan et al., 2009) and tested at passages 5–8. All measurements were performed either in this serum-containing medium or in serum-deprived [but insulin-transferrin-selenium (ITS)-supplemented] (Corning, Tewksbury, MA) medium, as indicated.

### Precision Cut Lung Slices

Lung slices were obtained from wild-type C57BL/6 mice (Jackson Laboratory, Bar Harbor, ME, USA) (Patel et al., 2017). Mice inhaled nebulized MCh (30 mg/ml) for 10 min followed by intratracheal (IT) treatment with either PEG400 (drug vehicle) or 5 mg/kg pitavastatin; treatments were repeated daily between postnatal day 15 and 20 (a total of 5 days). Mice were euthanized by cervical dislocation on postnatal day 21. Mouse lungs were infused with 1.5% low-melting-point agarose in Hanks' balanced salt solution, sectioned into 250 µm thick slices using a tissue slicer (VF-300; Precisionary Instruments, Greenville, NC), and cultured in Dulbecco's modified Eagle's medium (DMEM)/F12 medium (Thermo Fisher, Waltham, MA), as previously described (Aven et al., 2014). All mouse protocols were approved by the Institutional Animal Care and Use Committee at Brigham and Women's Hospital, Harvard Medical School.

Our approach is built around our previously published findings wherein we discovered that daily MCh nebulization between postnatal day 15 and 20 is sufficient to induce airway hypercontractility in mouse-derived PCLS at postnatal day 21 (Aven et al., 2014). Such airway hypercontraction is not driven by a change to ASM mass or airway inflammation but is due to the cholinergic stimulation of the ASM itself. Thus, any differences in airway constriction observed in PCLS obtained from pitavastatin and MCh co-exposed mice can be directly attributed to pitavastatin-induced changes to the ASM.

### Antibodies and Reagents

All chemicals were purchased from Sigma-Aldrich (St. Louis, MO), unless otherwise indicated. Antibodies for western blot analysis against phospho-MLC2 were purchased from Abcam (Cambridge, MA). Glyceraldehyde 3-phosphate dehydrogenase (GAPDH) antibody conjugated with human resource planning (HRP) was purchased from GeneTex (Irvine, CA). While pitavastatin was purchased from Santa Cruz Biotechnology Inc. (pitavastatin calcium, CAS 147526-32-7), the remaining statins (simvastatin, pravastatin) used in this study were purchased from Sigma-Aldrich (St. Louis, MO). Mevalonate was obtained through alkaline hydrolysis of mevalonolactone (Sigma-Aldrich, Catalog Number: M4667-1G). GGPP was purchased from Cayman Chemical (Ann Arbor, MI. Pre-designed small-interfering RNA (siRNA) against HMGCR, OptiMem, and Lipofectamine RNAiMax were purchased from Thermo Fisher (Waltham, MA). Human recombinant cytokines interleukin (IL)-13, IL-17, and TNFα were purchased from R&D Systems (Minneapolis, MN).

### Measurements of Airway Smooth Muscle Cell Contraction and Relaxation

Cellular force measurements were performed using the method of contractile force screening (Krishnan et al., 2009; Park et al., 2015; Yoshie et al., 2018). Briefly, this method utilizes a deformable substrate (Young's modulus = 3 kPa) prepared in a custom 96-well plate. Embedded close to the substrate surface is a single layer of fluorescent bead markers (diameter = 400 nm). Based on displacement of the markers, determined using an inverted fluorescence microscope (10x microscope objective, Leica DMI6000 B, Leica Microsystems, Buffalo Grove, IL), and knowledge of substrate stiffness, the ASM force map can be calculated using the method of Fourier transform traction cytometry (Butler et al., 2002), modified to the case of cell monolayers (Trepat et al., 2009). From each map, we computed the strain energy (i.e., the energy that is imparted to the substrate by the contractile cells, in pJ) to represent the average cellular contraction.

### Measurements of Airway Smooth Muscle Cytokine Secretion

Non-asthmatic human ASM cells were cultured to confluence for at least 72 h, and treated with either 2 µM pitavastatin (or vehicle) or 10 µM GGPP, and their combination, for a total of 72 h. For the final 18 h of this treatment period, cells were stimulated with a cytokine mixture comprising IL-13, IL-17, and TNFα (each at 10 ng/ml). Cells were harvested and cell-free media were subsequently collected. Expression of eotaxin-3 messenger RNA (mRNA) was analyzed by real-time (RT)-PCR, and cell-free media as supernatants were analyzed for secreted peptides IL-6 (Bio-Ocean, Shoreview, MN), IL-8 (Bio-Ocean, Shoreview, MN), and eotaxin-3 (Bio-Ocean, Shoreview, MN) using ELISA kits, as per manufacturer instructions. The sequence for the eotaxin-3 primers used were: Fwd: 5'-ACCTGCTGCTTCCAATACAGC-3' and Rev: 5'-CATAGCTTCGCACCCAGGTC-3'.

### Visualization of F-Actin Cytoskeletal Changes

ASM cells were fixed for 10 min with 3% formalin and then treated with phalloidin conjugated with Alexa Fluor 488 (1:500, Thermo Fisher, Waltham, MA) and Hoechst (Thermo Fisher, Waltham, MA) for an additional 30 min. Images were taken at a magnification of either 20x (Leica DMI6000 B, Leica Microsystems, Buffalo Grove, IL) or 40x (Zeiss LSM 880 confocal system, White Plains, NY). Imaging across all test groups was performed using the same laser power, gain, and offset conditions.

### Imposition of Mechanical Stretch

A single equi-biaxial stretch (10% magnitude, 4-s duration) was imposed using the method of Cell Mapping Rheometry (Krishnan et al., 2009).

### Western Blot Analysis

Cell lysates were prepared in ice-cold 1 mM ethylenediaminetetraacetic acid (EDTA) containing radioimmunoprecipitation assay (RIPA) buffer (Boston BioProducts, Ashland, MA) supplemented with phosphatase and protease inhibitors (Roche Diagnostics, Indianapolis, IN). Next, lysates were vortexed, sonicated, and centrifuged at 8,000 *g* for 10 min at 4°C, and supernatants were collected for additional testing. Electrophoresis, transfer, detection, and image acquisition were performed as described previously (Ghosh et al., 2012; Ghosh et al., 2015; Ghosh et al., 2016). Cell lysates were analyzed by western blot for total- and phospho-MLC2.

### Quantitative Real-Time PCR

Total RNA was extracted using the Quick-RNA Kit (Zymo Research, Irvine, CA). To transcribe the total RNA to cDNA, the qScript cDNA Synthesis Kit (Quantabio, Beverly, MA) was used. Next, we performed quantitative PCR (qPCR) reactions using the SYBR-green reaction mix (Bio-Rad, Hercules, CA) detected with the ABI 7500 Fast Real-Time PCR System (Thermo Fisher, Waltham, MA). Relative expression levels to ribosomal housekeeping controls (RPL-27) were determined using the comparative threshold method. Stable expression of RPL-27 was confirmed in all conditions. Total RNA for eotaxin-3 was extracted using Monarch Nucleic Acid Purification Kit (New England Biolabs, Ipswich, MA).

### Measurements of Bronchoconstriction in the Mouse Precision Cut Lung Slices

Airway lumen area was determined from bright field images by tracing a contour around the airway using the Magic Wand tool of the Fiji image analysis software (Schindelin et al., 2012). “% constriction” refers to the airway lumen area in response to 500 nM MCh normalized to the pre-treatment baseline value and expressed as a %.

### Measurements of Cellular and Precision Cut Lung Slices Toxicity

Toxicity measurements were performed in cultured human ASM cells using the RealTime-Glo™ Annexin V Apoptosis and Necrosis assay (Promega Inc., Madison, WI), as per the manufacturers' instructions. Toxicity measurements were performed in mouse PCLS using the Cell Titer 96^®^ AQ_ueous_ One Solution Reagent (MTS) (Promega, Madison, WI), as previously described (Watson et al., 2016). During MTS measurements in the PCLS, samples were incubated in DMEM/F12 medium without phenol red (Thermo Fisher, Waltham, MA).

### Statistical Analysis

Statistical analyses were performed using Prism version 8 (GraphPad software, San Diego, CA). Statistical comparisons were performed as a one-way or two-way ANOVA test followed by a *post-hoc* Tukey test, unless otherwise indicated (e.g., Student t test). *P-*values <0.05 (with two-way alpha) were considered statistically significant. All data unless otherwise speciefied are reported as the mean ± SEM (standard error of the mean).

## Results

### Pitavastatin Reduces Basal- and Agonist-Induced Airway Smooth Muscle Contraction

**Figure 1:**
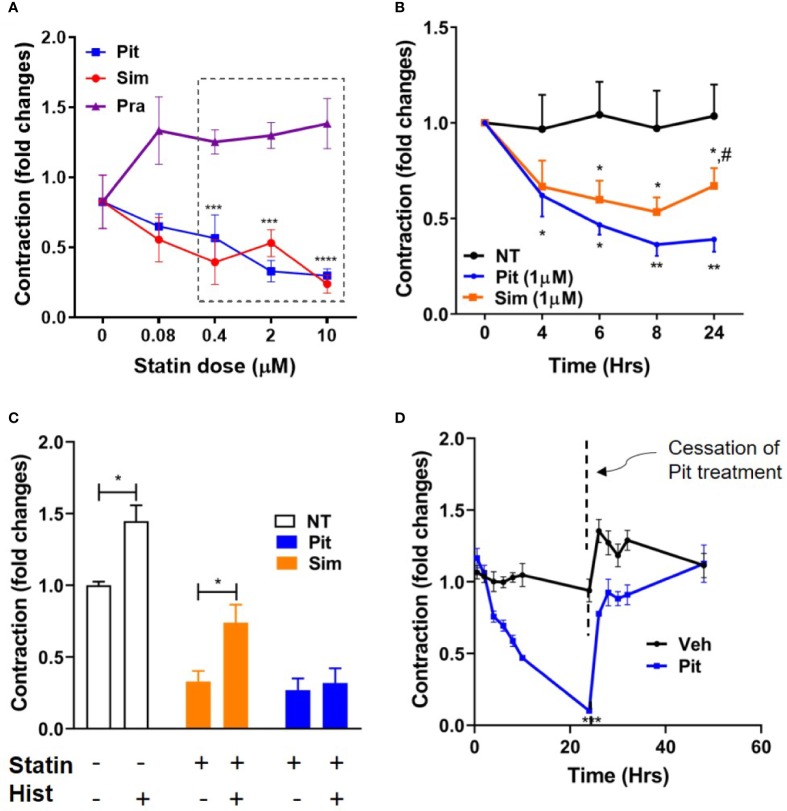
Pitavastatin inhibits basal- and histamine-induced airway smooth muscle (ASM) contraction. **(A)** As compared to no treatment (0 µM), statistically significant reductions in contraction occurred at 24 h as follows: pitavastatin (Pit) at 0.4, 2, and 10 µM and simvastatin (Sim) at 0.4 and 10 µM. Under similar experimental conditions, pravastatin had no effect on ASM cell contraction. **(B)** While both 1 µM Sim and 1 µM Pit reduced ASM contraction time-dependently compared to no-treatment (NT), Pit was significantly more efficacious than Sim at 24 h (indicated by ^#^). **(C)** Compared to NT, both 0.4 μM Pit and 0.4 μM Sim reduced basal ASM contraction to a similar extent after 24-h of treatment. However, while 0.4 μM Pit inhibits histamine (Hist)-induced ASM contraction, 0.4 μM Sim does not. Statistical comparison was performed using the student t-test. **(D)** The force inhibitory effects of 1 μM Pit was reversed when the wells were resuspended with media without Pit. For **(A–D)**, all experiments were performed using one non-asthmatic primary human ASM donor line. **(A, B, D)** were performed in serum [10% fetal bovine serum (FBS)]-containing media conditions while **(C)** was performed under serum-deprived media conditions, imposed for 24 h. Serum deprivation was imposed to enhance the ability of the ASM to contract to histamine (Halayko et al., 1999). In all graphs, ASM contraction is plotted as fold change to the pre-treatment baseline value at 0 h. p-values: *,^#^p < 0.05; **p < 0.01; ***p < 0.001; ****p < 0.0001. For each group, n=4–8 separate wells per condition.

Using contractile force screening (Park et al., 2015; Yoshie et al., 2018; Yoshie et al., 2019), we discovered that while the lipophilic statins pitavastatin and simvastatin reproducibly inhibited basal ASM contraction, the hydrophilic statin pravastatin did not (**Figure 1A**). Additional time-course measurements revealed that the inhibitory effect of pitavastatin was greatest at 24 h (**Figure 1B**). Furthermore, when compared to no treatment (NT), while both pitavastatin and simvastatin (at 0.4 µM each) reduced basal ASM contraction to a similar extent, pitavastatin uniquely inhibited additional histamine-induced ASM contraction (**Figure 1C**). Finally, the force inhibitory effects of pitavastatin was reversible after cessation of treatment (**Figure 1D**).

**Figure 2:**
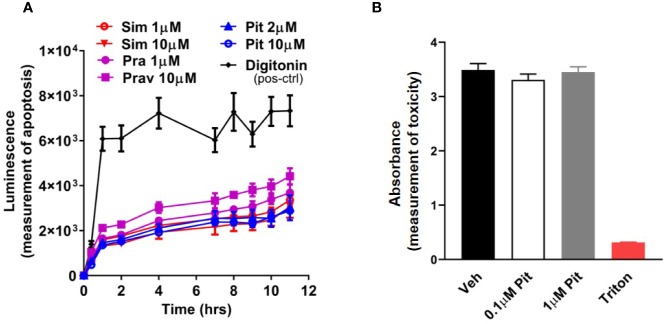
Pitavastatin is non-toxic in cells and precision cut lung slices (PCLS). **(A)** Statin treatment did not induce apoptosis in airway smooth muscle (ASM) cells derived from one human non-asthmatic donor. Digitonin (50 µg/ml) was used as a positive control. The experiment was performed in serum [10% fetal bovine serum (FBS)]-containing media conditions. n=3 wells per condition; n's indicate the number of separate wells of ASM monolayers. **(B)** As compared to no treatment (0 µM), no reductions in viability were observed in mouse PCLS treated with pitavastatin. 0.01% Triton treatment for 2 h is included as a positive control. For each of the 0, 0.1, and 1 µM pitavastatin treatment groups, we used n=25 slices that were obtained from four mice, with three to eight slices obtained per mouse. For the triton group, we used n=4 slices obtained from two mice, with two slices per mouse.

Although safe and generally well-tolerated, a potential risk with excessive statin use is muscle toxicity (Furberg and Pitt, 2001; Alsheikh-Ali et al., 2005; Ward et al., 2019). Given this knowledge, we examined dose- and compound-dependent toxicity of statins on ASM. We did not detect any cellular (**Figure 2A**) or murine PCLS (**Figure 2B**) toxicity.

**Figure 3:**
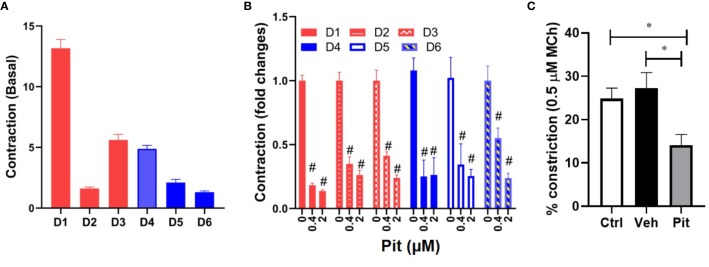
Pitavastatin inhibits basal contraction of asthmatic human airway smooth muscle (ASM) cells and methacholine (MCh)-induced constriction of murine precision cut lung slices (PCLS). **(A)** We observed heterogeneity in basal ASM contraction in both asthmatic (D1–D3) and non-asthmatic (D4–D6) donors. **(B)** Across both asthmatic and non-asthmatic donor cells, pitavastatin dose-dependently inhibited ASM contraction (#p < 0.0001 compared to 0 µM treatment). Cells from each donor were tested using n=4–8 separate wells. All cellular experiments were performed in serum [10% fetal bovine serum (FBS)]-containing media conditions. **(C)** When PCLS derived from neonatal mice pre-exposed to MCh alone (Ctrl), MCh+vehicle (Veh), or MCh+pitavastatin (Pit) were treated with 500 nM MCh, the airways of the Pit group constricted significantly less (Ctrl=24.9%, Veh=27.2%, Pit=14.2%, *p < 0.05). For each group, we used n=10–13 PCLS that were obtained from 2 to 3 mice.

To clarify disease-relevance, we evaluated the effects of pitavastatin in ASM cell lines obtained from asthmatic human donor lungs. We observed heterogeneity in basal ASM contraction in both asthmatic (D1–D3) and non-asthmatic (D4–D6) donors (**Figure 3A**). Regardless of donor (indicated as D1–D6) or disease status (indicated as non-asthmatic *vs.* asthmatic), pitavastatin treatment for 24 h dose-dependently inhibited ASM contraction (**Figure 3B**). The percentage (%) of force inhibition was not statistically different between asthmatic and non-asthmatic donors. Finally, we turned to mouse PCLS which were obtained from neonatal mice after they were exposed to nebulized MCh in the presence (or absence) of pitavastatin treatment (5 mg/kg IT). Upon treatment with 500 nM MCh, airways of the pitavastatin-pretreated group constricted significantly less (**Figure 3C**).

### Pitavastatin Inhibits the Airway Smooth Muscle Cytoskeleton *via* a Mevalonate- and Geranylgeranyl Pyrophosphate-Dependent Mechanism

**Figure 4:**
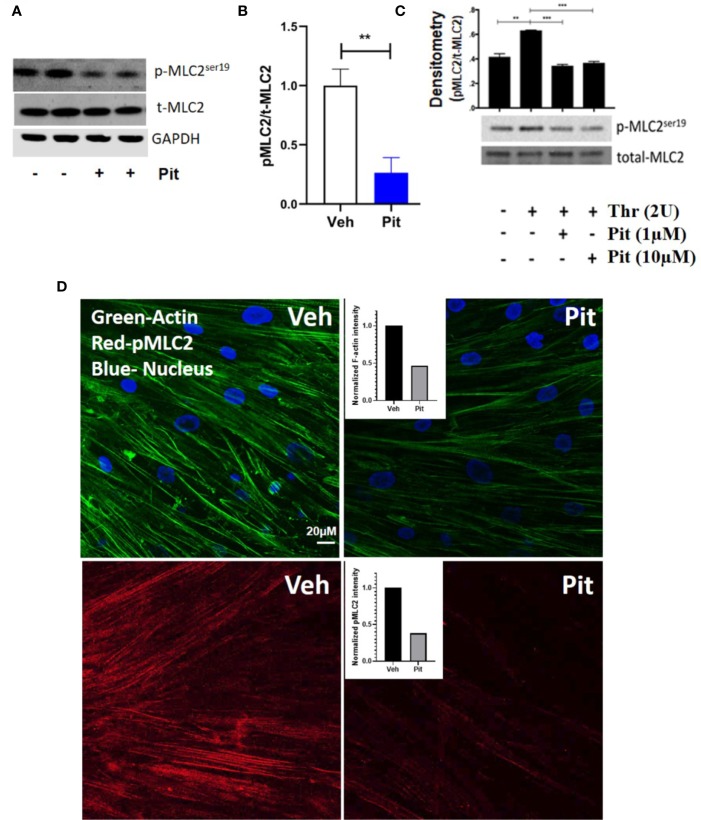
Pitavastatin inhibits the airway smooth muscle (ASM) cytoskeleton. ASM cells from a non-asthmatic human donor lung were grown in serum [10% fetal bovine serum (FBS)]-containing media in the presence of drug-vehicle (Veh) or 1 μM pitavastatin (Pit) for 24 h. **(A–C)** Pit treatment significantly reduced basal phospho-MLC2 (pMLC2) expression, but not total-MLC2. Glyceraldehyde 3-phosphate dehydrogenase (GAPDH) was used as a protein loading control. Pit treatment also significantly reduced thrombin (2 U/ml, 30 min)-induced pMLC2 enhancement. **(D)** Immunostaining measurements revealed that pitavastatin significantly reduced F-actin (green) and pMLC2 (red) expression. For these representative images, F-actin and pMLC2 intensities were quantified using the “integrated density” measurement in ImageJ and normalized to Veh. Scale bar=50 μm. p-values: **p < 0.01; ***p < 0.001.

Pitavastatin treatment for 24 h inhibited basal MLC2 phosphorylation (**Figures 4A, B**) and the F-actin cytoskeleton (**Figure 4D**). Beyond this agonist-independent effect, pitavastatin also inhibited thrombin-induced phospho-MLC2 enhancement (**Figure 4C**).

**Figure 5:**
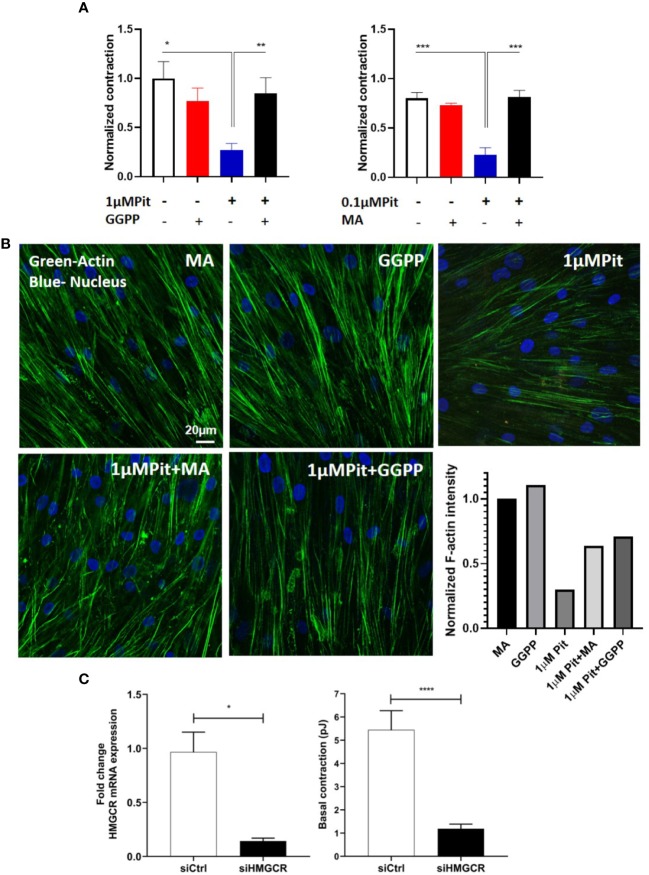
Pitavastatin Inhibits the airway smooth muscle (ASM) cytoskeleton *via* a mevalonate (MA)- and geranylgeranyl pyrophosphate (GGPP)-dependent mechanism. ASM cells obtained from a non-asthmatic human donor lung were grown in serum [10% fetal bovine serum (FBS)]-containing media in the additional presence of one of the following five supplements: drug-vehicle, 0.1 “or” 1 μM pitavastatin (Pit), 10 μM GGPP, 1 μM Pit+10 μM GGPP, 100 μM MA, 0.1 μM “or” 1 μM Pit+100 μM MA for 24 h. **(A)** While Pit alone inhibited basal ASM contraction, co-treatment with MA or GGPP abrogated Pit-induced ASM force inhibition. **(B)** While Pit alone inhibited ASM F-actin expression, co-treatment with GGPP or MA abrogated Pit-mediated F-actin inhibition. Scale bar = 20 μm. For these representative images, F-actin intensity was quantified using the “integrated density” measurement in ImageJ and normalized to the mevalonate (MA) group. **(C)** Silencing 3-hydroxy-3-methylglutaryl-coenzyme A reductase (HMGCR) expression in the non-asthmatic human ASM donor line used in **(A, B)** for 48 h significantly reduced basal ASM contraction. n=12 separate well of ASM monolayer per condition. All experiments were performed in serum (10% FBS)-containing media conditions. p values: *p < 0.05; **p < 0.01; ***p < 0.001; ****p < 0.0001.

The ASM force reduction and F-actin ablation by pitavastatin was blunted with MA and GGPP co-treatment (**Figures 5A, B**). Finally, when HMGCR gene expression was silenced in ASM cells from a non-asthmatic human donor, ASM cellular contraction was significantly inhibited (**Figure 5C**). These data suggest a central role for the MA pathway in the force- and cytoskeletal-inhibitory effects of pitavastatin.

### Pitavastatin Augments the Airway Smooth Muscle-Relaxing Effects of a Simulated Deep Breath

**Figure 6:**
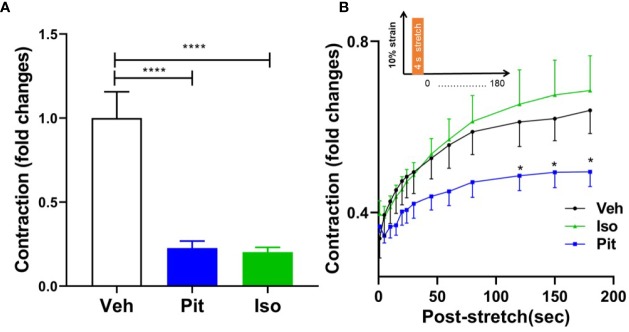
Pitavastatin potentiates the airway smooth muscle (ASM) relaxation effect of a simulated deep breath, a beneficial effect of pitavastatin that is absent for isoproterenol. **(A)** As compared to untreated controls (n=7), pre-treatment with 1 µM Pit (24 h) (n=7) or 10 µM isoproterenol (Iso, 30 min) (n=6) significantly inhibited basal ASM contraction. Shown are contraction values normalized to the untreated control group. **(B)** In response to a subsequent single stretch-unstretch maneuver that mimics a deep breath (10% magnitude, 4-s duration, see inset), the ASM cell promptly and dramatically ablates its contraction. The forces then subsequently recover over 180 s. While force ablation was similar across all three groups, the subsequent force recovery was significantly inhibited by Pit treatment. The n's indicate the number of separate wells of ASM monolayers. The experiment was performed in serum [10% fetal bovine serum (FBS)]-containing media conditions. P-values: *p < 0.05; ****p < 0.0001.

Treatment with 1 µM pitavastatin for 24 h or 10 µM isoproterenol for 30 min reduced ASM force to the same extent (**Figure 6A**). However, in response to a superimposed transient stretch, only pitavastatin potentiated stretch-induced ASM relaxation (**Figure 6B**). This shows a unique property of pitavastatin not observed with the β2-agonist isoproterenol.

### Pitavastatin Attenuates Cytokine Induced Pro-Inflammatory Cytokine/Chemokine Secretion in Airway Smooth Muscle

**Figure 7:**
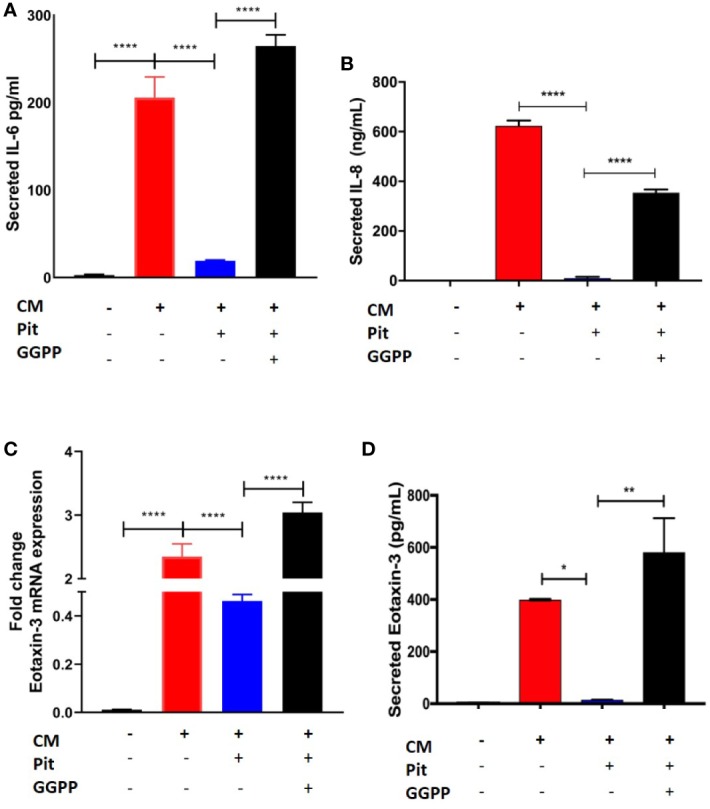
Pitavastatin inhibits airway smooth muscle (ASM) inflammation by a geranylgeranyl pyrophosphate (GGPP)-dependent mechanism. ASM cells obtained from a non-asthmatic human donor lung were grown in serum [10% fetal bovine serum (FBS)]-containing media for at least 72 h and were treated with either drug-vehicle, 2 μM Pit, or 10 μM GGPP for a total of 72 h. During the final 18 h of treatment, cells were exposed to a cytokine mixture (CM) comprising interleukin (IL)-13, IL-17, and TNFα, at 10 ng/ml each. **(A–D)** While Pit alone inhibited CM-induced IL-6, IL-8, and eotaxin-3 production, co-treatment with GGPP abrogated these effects, confirming a GGPP-dependent mechanism. GGPP alone or CM+GGPP had no inducing or inhibiting effect on these cytokines/chemokines (data not shown). p values: *p < 0.05; **p < 0.01; ****p < 0.0001.

Exposure of non-asthmatic human ASM cells to a cytokine mixture (CM) containing IL-13, IL-17, and TNFα (each at 10 ng/ml) induced IL-6, IL-8, and eotaxin-3 production from ASM cells, as measured by RT-PCR and cytokine-specific ELISA (**Figures 7A–D**). Pre-treatment with pitavastatin blunted these effects in a GGPP-dependent manner. This confirms that the MA pathway mediates the production of pro-inflammatory cytokines from human ASM.

## Discussion

The principal finding of this study is that pitavastatin inhibits key components of the cytoskeletal machinery of ASM contraction including MLC2 phosphorylation and F-actin formation. This confers unique bronchoprotective effects, especially the potentiation of stretch-induced ASM relaxation.

Statins differed in their inhibitory potency of ASM contraction and did not adhere to the notion of a “class effect” as would be expected of HMGCR inhibitors. For example, the hydrophilic statin, pravastatin did not inhibit ASM contraction while the moderately lipophilic statin, pitavastatin, and the highly lipophilic statin, simvastatin significantly relaxed ASM (**Figure 1A**). However, the effect of pitavastatin was more prolonged (**Figure 1B**), and also more efficacious at inhibiting histamine-induced ASM contraction (**Figure 1C**) compared to equimolar simvastatin. This indicates that the degree of lipophilicity alone may not fully dictate statin efficacy with respect to ASM relaxation, where other factors may be at play. These factors might include chemical parameters such as absorption, membrane permeability, compound metabolism or some combination thereof. In summary, our studies were able to differentiate statins by their ASM relaxing effects, and identified a novel candidate for further study: pitavastatin.

**Figure 8:**
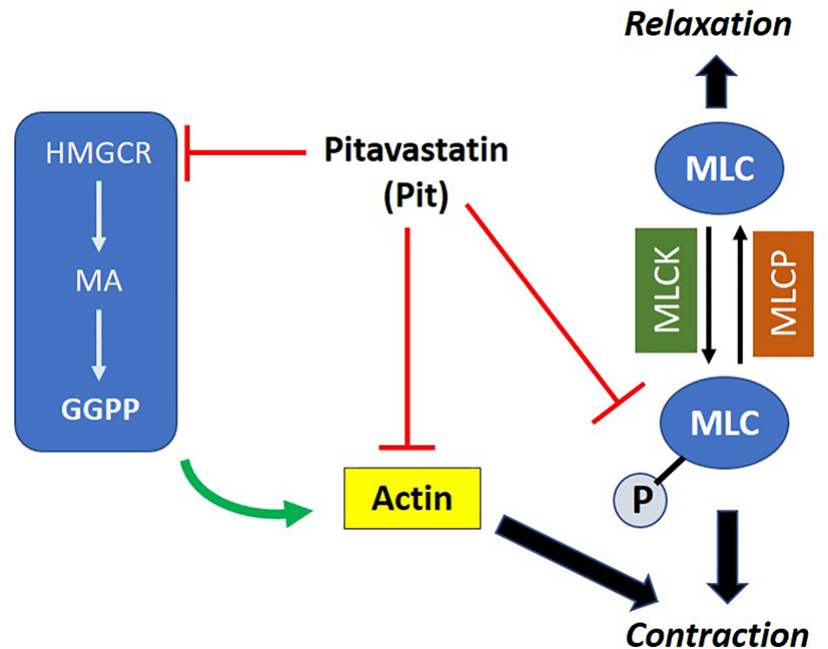
Proposed mechanism of action of pitavastatin: pitavastatin inhibits components of the airway smooth muscle (ASM) cytoskeletal apparatus including myosin light chain (MLC) and F-actin. The inhibition of F-actin occurs by a geranylgeranyl pyrophosphate (GGPP)-dependent mechanism. Pitavastatin depletes the pool of available intracellular GGPP by inhibiting 3-hydroxy-3-methylglutaryl-coenzyme A reductase (HMGCR). This schema explains, at least in part, the molecular mechanism by which pitavastatin inhibits human ASM contraction.

We hypothesized that pitavastatin induces ASM relaxation by ablating the ASM contractile apparatus. In accordance, we observed decreases in MLC2 phosphorylation (**Figures 4A–C**) and F-actin levels (**Figure 4C**). We also discovered the biochemical mechanism mediating these statin effects involves the MA pathway, and more specifically, the isoprenoid lipid GGPP (**Figures 5** and **8**). This suggests that the basic mechanism involves alterations in the metabolism of intracellular MA, and thus, serves to further illustrate the uniqueness of this pharmacologic approach as a potential new therapy to treat ASM contraction.

Pitavastatin may inhibit F-actin levels by simply reducing its gene expression, or by preventing the regulatory steps that lead to F-actin production, polymerization, or arrangement. For example, statins are known inhibitors of histone deacetylases (HDACs) [e.g., (Singh et al., 2016)]. By inhibiting HDAC8 in ASM, pitavastatin might be attenuating actin polymerization and subsequent ASM contraction (Li et al., 2014; Tang, 2015). Pitavastatin is likely to also alter how actin is anchored to the cell plasma membrane *via* adhesion protein complexes (Gunst and Zhang, 2008). Because statins reduce plasma membrane cholesterol levels (and disintegrate lipid rafts), this makes the membrane less rigid (Forsyth et al., 2012; Sheikh-Hasani et al., 2018). This could destabilize or reduce actin filament anchoring in the membrane thereby weakening its connection to adhesion complexes. Statins are known to reduce integrin expression (Takeda et al., 2007), and this is another mechanism whereby connection to the cytoplasmic tails could be disrupted. Finally, statins may regulate protein kinase pathways such as Ste20-like kinase (SLK) (Wang et al., 2020) and polo-like kinase 1 (Plk1) (Li et al., 2016) and thus induce structural transformation of the actin-cytoskeletal apparatus that is responsible for force transmission (Seow and An, 2020).

The role of small monomeric GTPases with respect to the cytoskeletal signaling dynamics involved in ASM contraction are multi-faceted (Zhang et al., 2012; Zhang et al., 2018). For example, the GTPase action of RhoA leads to paxillin tyrosine phosphorylation which leads to paxillin-N-WASp mediated actin formation (Zhang et al., 2012). Cdc42, another Rho family GTPase leads to N-WASp and Arp2/3 complex activation which then leads to actin polymerization (Zhang et al., 2018). By depleting cellular GGPP (**Figure 5**), pitavastatin reduces levels of both geranylgeranylated RhoA and Cdc42. Unprenylated RhoA and Cdc42 cannot anchor in cell plasma membranes, and thus, cannot participate in the activation of protein complexes, as per above. This is one mechanism whereby a statin could inhibit tyrosine phosphorylation of important complex proteins such as paxillin and thus indirectly inhibit actin polymerization. We have already begun to investigate the effects of pitavastatin on RhoA and ROCK, including ROCK1 and ROCK2 phosphorylation in ASM cells, and examine how this compares to known rho-associated kinase (ROCK).

In addition to RhoA/ROCK, Abelson tyrosine kinase (Abl) plays an important role in regulating ASM contraction and proliferation *in vitro* (Tang, 2015). While no studies have yet linked Abl to statins and ASM contraction changes, other studies in different cell systems indicate that statins decrease the expression of other tyrosine kinases such as Focal adhesion kinase (FAK) (Rentala et al., 2013). The same could be occurring in ASM cells with pitavastatin, but this remains to be determined.

Pitavastatin augmented stretch-induced ASM force relaxation (**Figure 6B**). When compared to other emerging targets for potentiating stretch-induced ASM relaxation including zyxin (Rosner et al., 2017), cofilin (Lan et al., 2018), actin-myosin-actin connectivity (Lavoie et al., 2009), or cytoskeletal signaling elements (Cleary et al., 2013), the practical advantage of targeting the MA pathway in ASM using pitavastatin is that it is expected to have a shorter path to development (as an oral or inhaled agent) than other new chemical compounds whose safety and toxicity profiles in humans are unknown.

We made three simplifying assumptions in our experimental plan. First, to model AHR and its treatment, our studies were limited to PCLS obtained from mice that were pre-exposed to MCh ± pitavastatin. But given that pitavastatin inhibits force-generation even in non-asthmatic human ASM cells, a logical next step is to examine a potential bronchoprotective effect of pitavastatin in naïve mouse PCLS. Second, in order to isolate statin effects on ASM and clarify the mechanism of action, we limited our investigation of ASM contraction to inflammation-independent settings. Furthermore, most mechanistic experiments were limited to ASM cells obtained from one non-asthmatic donor. An important future direction would be to examine the ability of pitavastatin to relax ASM in allergen-induced lung inflammation (Wu et al., 2017). Importantly, these experiments should encompass multiple non-asthmatic and asthmatic donors in order to critically evaluate the issue of donor-to-donor variability. Finally, a key finding of our studies is that pitavastatin has preventative benefit. That is to say, even in the absence of histamine and MCh stimulation, it reduces ASM contractile tone by inhibiting the ASM cytoskeleton. It remains to be seen whether pitavastatin can be an effective preventative strategy for the development of AHR in naïve mice/non-asthmatic subjects, or those with milder *versus* more severe forms of asthma or bronchospasm.

In conclusion, using non-asthmatic and asthmatic human ASM cells, and murine PCLS, we provide foundational evidence that pitavastatin inhibits basal- and agonist-induced ASM contraction. Mechanistically, this entails a molecular pathway that more directly targets the ASM cytoskeleton (**Figure 8**) than existing bronchodilators that are largely focused on the indirect action of ASM-receptor mediated changes. Functionally, this manifests in enhancing transient stretch-induced ASM relaxation. Finally, pitavastatin attenuates pro-inflammatory ASM cytokine secretion. Taken together, these findings make pitavastatin an attractive candidate for further development as a bronchodilator therapy.

## Data Availability Statement

All relevant datasets generated for this study are included in the article/supplementary material.

## Ethics Statement

All mouse protocols were approved by the Institutional Animal Care and Use Committee at Brigham & Women's Hospital, Harvard Medical School.

## Author Contributions

RL performed TFM measurements in ASM cells. RL, NN, AZ and KC performed molecular measurements in ASM cells. SR-M, YB, and XA performed mouse PCLS measurements. SP, AZ, RK, and CG planned the study, analyzed the data, and wrote the manuscript.

## Funding

This work was supported, in part, by 1R03AI139648-01A1 (AZ), and U.C. Davis PI Bridge Fund (AZ).

## Conflict of Interest

RK, AZ, SP, and CG are founders of InStatin, Inc., a private company that has an interest in this research.

The remaining authors declare that the research was conducted in the absence of any commercial or financial relationships that could be construed as a potential conflict of interest.
